# Active surveillance of papillary thyroid microcarcinomas in South America: Are we ready?

**DOI:** 10.20945/2359-3997000000180

**Published:** 2019-10-03

**Authors:** Debora Lucia Seguro Danilovic, João Roberto M. Martins, Ana Luiza Maia

**Affiliations:** 1 Hospital das Clínicas Faculdade de Medicina Universidade de São Paulo São Paulo SP Brasil Laboratório de Endocrinologia Celular e Molecular (LIM25), Hospital das Clínicas , Faculdade de Medicina da Universidade de São Paulo (HCFMUSP), São Paulo , SP , Brasil; 2 Instituto do Câncer do Estado de São Paulo Faculdade de Medicina Universidade de São Paulo São Paulo SP Brasil Instituto do Câncer do Estado de São Paulo , Faculdade de Medicina da Universidade de São Paulo (FMUSP), São Paulo , SP , Brasil; 3 Departamento de Medicina Escola Paulista de Medicina Universidade Federal de São Paulo São Paulo SP Brasil Laboratório de Endocrinologia Molecular e Translacional, Disciplina de Endocrinologia e Metabologia, Departamento de Medicina , Escola Paulista de Medicina , Universidade Federal de São Paulo (UNIFESP/EPM), São Paulo , SP , Brasil; 4 Unidade de Tireoide Hospital de Clínicas de Porto Alegre Universidade Federal do Rio Grande do Sul Porto Alegre RS Brasil Unidade de Tireoide , Hospital de Clínicas de Porto Alegre , Universidade Federal do Rio Grande do Sul (UFRGS), Porto Alegre , RS , Brasil

Active surveillance is defined as a strategy for treating a medical condition that involves a period of waiting and regular testing, rather than immediate treatment, such as surgery. In previous years, active surveillance (AS) of papillary thyroid microcarcinomas (mPTC; tumors with less than 1.0 cm) has been the focus of discussion worldwide, notably in the context of the epidemic of thyroid carcinomas diagnosed by neck imaging exams (1). This practice was initially proposed in 1993 by Miyauchi and cols. and, recently, the Japanese group reported their experience in AS of 1235 patients diagnosed with mPTC at Kuma Hospital (2). Between 1993 and 2011, patients with low-risk mPTC chose observation without immediate surgery. They were followed periodically with ultrasound examinations. It was set three parameters for the evaluation of PTMC progression: (i) size enlargement, (ii) novel appearance of lymph-node metastasis, and (iii) progression to clinical disease (tumor size reaching 12 mm or larger, or novel appearance of nodal metastasis). The proportion of patients with mPTC progression was lowest in the old patients and highest in the young patients. The reported outcomes are excellent: 8% tumor enlargement greater than 3 mm and 3.8% of novel lymph nodes metastases in a 10 years follow-up period. Such promising experiences have also been recently reported by other groups, mainly in the US (3) and Korea (4).

In this issue, two articles discuss the overdiagnosis of thyroid nodules and the active surveillance in mPTC as an alternative treatment in South American patients. In an interesting study, Smulever and Pitoia (5) reported their experience in AS of mPTC in a Latin American reference center. The authors have proposed AS for those patients with mPTC who underwent fine needle aspiration biopsy (FNAB) procedure with Bethesda category V and VI results. The inclusion criteria were: the presence of a single nodule; tumor size ≤ 1.5 cm in maximal diameter; absence of clinical or radiological evidence of extrathyroidal extension, invasion of local structures, regional or distant metastasis. The authors pointed out that only 25% of 136 patients eligible for AS accepted this approach, and about 10% of these subjects abandoned AS, mainly due to their anxiety about the disease. However, the favorable outcomes of those patients that did not undergo surgery were similar to those previously reported aforementioned, with tumor enlargement occurring in only 17% of the cases and no lymph or distant metastases being diagnosed in a median 4.6 years of follow-up. There are no features that differentiate patients with stable tumors from those with increased tumor (≥ 3 mm).

Yet, in the other article, a group of Brazilian experts states a series of recommendations to avoid overdiagnosis of thyroid nodules and overtreatment of mPTC (6). The authors suggested FNAB of very suspicious nodules ≤ 1 cm only if age < 40 years, presence of extrathyroidal extension on ultrasonography (US), nodule adjacent to the trachea or recurrent laryngeal nerve, multiple suspicious nodules, presence of suspicious lymph nodes or known distant metastases. Clinical and imaging follow-up is recommended if the patient is not submitted to FNAB. The same criteria to preclude FNAB of nodules ≤ 1 cm was suggested to select mPTC to AS. AS would consist of periodic clinical examination, serum TSH measurement, US every 6 months in the first year and then annually. The authors highlighted that patient should agree with the protocol.

Are we ready to implement AS in South America? In order to effectively implement active surveillance of mPTC, some aspects should be considered. Firstly, physicians should be confident about the safety of AS as a treatment alternative to immediate surgery to encourage patients to this conservative approach. Indeed, acceptance of AS at Kuma Hospital increased gradually over a 24 years period, from 30% in 1993-1997 to 88% in 2014-2016 (7). Ito and cols. reported that up to 2007 most patients were seen only by surgeons but, since then, therapeutic strategies were increasingly determined by endocrinologists, who adopted AS more frequently, particularly after publications regarding successful AS follow-up. This increase in the acceptance of AS might also be influenced by the features of the population under study, proximity to the Thyroid Cancer Center and education sessions provided to patients in a specialized setting (8).

Furthermore, adequate conditions to perform AS must be assured. Some argue that adverse events related to thyroidectomy, especially when performed by inexperienced surgeons, would favor the avoidance of surgery in low-risk cancer. Life-long consequences of permanent hypothyroidism, hypoparathyroidism, and lesion of recurrent laryngeal nerve increase not only health care costs, but also compromise the patient’s quality of life (9). On the other hand, the safety of AS is based on periodic follow-up evaluations, including carefully performed US. Additionally, it is mandatory that patients understand the importance and commit to the conditions to AS. Public health care system must guarantee medical support to the follow-up evaluation in order to identify tumor progression and metastases. As suggested by Rosario and cols. (6), lobectomy would be a reasonable approach if the above conditions are not assured.

Considering disparity in healthcare access in Brazil, academic reference centers should lead the implementation of AS in patients with mPTC. As in Kuma Hospital and Memorial Sloan Kettering Cancer Center (3), it could be initiated as institutional protocols. Nationwide implementation will depend on continuous physicians and patients’ proper medical education and the assurance of adequate health care support.
